# Development of a whole‐cell high‐throughput phenotypic screen to identify inhibitors of mycobacterial amino acid biosynthesis

**DOI:** 10.1096/fba.2018-00048

**Published:** 2019-01-22

**Authors:** Christopher Burke, Katherine A. Abrahams, Emily J. Richardson, Nicholas J. Loman, Carlos Alemparte, Joel Lelievre, Gurdyal S. Besra

**Affiliations:** ^1^ School of Biosciences University of Birmingham Birmingham UK; ^2^ Diseases of the Developing World, GlaxoSmithKline Madrid Spain

**Keywords:** drug discovery, high‐throughput screening, tuberculosis

## Abstract

Anti‐tubercular drug discovery continues to be dominated by whole‐cell high‐throughput screening campaigns, enabling the rapid discovery of new inhibitory chemical scaffolds. Target‐based screening is a popular approach to direct inhibitor discovery with a specified mode of action, eliminating the discovery of anti‐tubercular agents against unsuitable targets. Herein, a screening method has been developed using *Mycobacterium bovis *BCG to identify inhibitors of amino acid biosynthesis. The methodology was initially optimized using the known branched‐chain amino acid biosynthetic inhibitors metsulfuron‐methyl (MSM) and sulfometuron‐methyl (SMM), and subsequently, whole genome sequencing of resistant mutants and the use of over‐expressor strains confirming their mode of action. The GlaxoSmithKline compound library of small molecule inhibitors with known activity against *Mycobacterium tuberculosis* was then used to validate the screen. In this paper, we have shown that media supplementation with amino acids can rescue *M bovis *BCG from known amino acid synthesis inhibitors, MSM and SMM, in a pathway specific manner. The therapeutic potential of amino acid biosynthesis inhibitors emphasizes the importance of this innovative screen, enabling the discovery of compounds targeting a multitude of related essential biochemical pathways, without limiting drug discovery toward a single target.

## 1 INTRODUCTION

*Mycobacterium tuberculosis*(*Mtb*), the causative agent of tuberculosis (TB), is the world's most successful pathogen, with a higher mortality rate than HIV.^1^ In 2016, there were an estimated 600 000 cases of both multi‐drug resistant TB (MDR‐TB) and rifampicin‐resistant TB (RR‐TB), with 153 000 new cases detected in 2016.^2^ With only 54% of MDR‐TB and RR‐TB successfully treated, there is a need to develop new drugs with unique modes of action.^2^

Amino acid biosynthesis is an attractive and validated target for anti‐mycobacterial drugs.^3^, ^4^ Since the advent of whole genome sequencing (WGS), the genes involved in the synthesis of all 20 amino acids in *Mtb* have been identified, enabling the deconvolution of their biosynthetic pathways.^5^ A number of studies have demonstrated that the enzymes involved in these pathways are essential for survival in nutrient‐limited environments, such as in the host.^6^, ^7^ For example, auxotrophs for proline, tryptophan,^8^ lysine,^9^ arginine,^10^ methionine,^11^ cysteine,^12^ and branched‐chain amino acids,^6^, ^13^, ^14^ have demonstrated attenuation in murine infections. The essentiality of other amino acid biosynthetic pathways including histidine and the aromatic amino acids phenylalanine, tyrosine and tryptophan has also been demonstrated.^15^, ^16^, ^17^ Many of these amino acid biosynthetic pathways have been suggested as attractive targets for drug development due to their importance with bacterial growth, and the fact that many of the above amino acids are classified as essential in humans and lack human orthologs, thereby exhibiting selective toxicity.

Herein, we have developed a method to identify inhibitors of amino acid biosynthesis using a whole‐cell phenotypic screening approach. We have validated this method using two known inhibitors of branched‐chain amino acid synthesis, metsulfuron‐methyl (MSM) and sulfometuron‐methyl (SMM), and screened an anti‐tubercular compound library from GSK which identified an inhibitor of tryptophan synthesis. This targeted approach not only accelerates mode of action studies, but also focuses inhibitor discovery toward a suitable target.

## 2 METHODS

### 2.1 Growth conditions for *Mycobacterium bovis* BCG and *Mycobacterium smegmatis*

*Mycobacterium bovis *BCG and *M smegmatis* cultures were grown in cell culture flasks in Middlebrook 7H9 media supplemented with 10% v/v oleic acid, albumin, dextrose, and catalase (OADC), 0.2% v/v glycerol and 0.05% v/v Tween 80. *M bovis* BCG culture strains were incubated at 37°C with 5% CO_2_. *M smegmatis *culture strains were incubated at 37°C with shaking at 180 rpm. Cells containing plasmids pVV16k (Kanamycin resistance marker) and pMV261a (Apramycin resistance marker) were grown containing 25 μg/mL of the appropriate antibiotic in culture.

### 2.2 MIC determination in liquid media

The liquid MIC of MSM and SMM for *M bovis* BCG and *M smegmatis* were conducted in Greiner black flat‐bottomed 96‐well plates. Mid‐log (OD_600nm _0.4‐0.8) cells were diluted to 1 × 10^6 ^cells/mL in Middlebrook 7H9 culture medium and were added to each well along with increasing concentrations of drug. MSM and SMM were made up in 100% dimethyl sulfoxide (DMSO) and added at a final concentration of 1% DMSO. A 1% DMSO only positive control and a 1 μmol/L rifampicin negative control were also used. A media only liquid barrier was maintained around the entirety of the test wells to minimize evaporation. The 96‐well plates were incubated 7 days for *M bovis *BCG and 24 hours for *M smegmatis* at 37°C with 5% CO_2_. After incubation, cell survival was monitored using resazurin.^18^ Briefly, 30 μL of 0.05% resazurin and 12.25 μL of 20% tween 80 were added to every well and incubated for a further 24 hours for *M bovis *BCG and 2 hours for *M smegmatis*. Plates were then read using a BMG plater reader at excitation wavelength 544 nmol/L and detection wavelength 590 nmol/L. Percentage survival was calculated as per the equation $\%\text{Survival}=\left(\frac{x-\hat{n}}{\hat{p}-\hat{n}}\right)\times 100$. Where x equals the experimental value, $\hat{p}$ equals the mean of the positive controls, and $\hat{n}$ equals the mean of the negative controls.

### 2.3 Whole cell rescue of MSM and SMM inhibition by amino acid supplementation

Wild‐type (WT) *M bovis *BCG was grown to mid‐log density (OD_600nm _0.4‐0.8) and diluted into 2 × Middlebrook 7H9 to 2 × 10^6^ cells/mL. Mixtures of leucine, isoleucine and valine, and of all 20 amino acids, or 20 mixtures of 19 amino acids (each mixture lacking a different amino acid) were made at 2× required concentration (stated where appropriate) and adjusted to pH 7.4. MSM and SMM were added into Greiner black flat‐bottomed 96‐well plates. Cells and amino acid mixtures were mixed in equal volumes to attain 1 x 7H9 concentration with 1 × 10^6^ cells/mL. 7H9 with cells and amino acids were then added to each well up to 100 μL at a final concentration of up to 2% DMSO. The 96‐well plates were then incubated for 7 days at 37°C with 5% CO_2_. After incubation, cell survival was monitored using the resazurin assay as described.^18^

### 2.4 Spontaneous resistant mutant generation and WGS

*Mycobacterium bovis* Bacillus Calmette‐Guérin (BCG) resistant mutants were generated by plating 1 × 10^8^ mid‐log cells on solid media (7H11 agar with 10% OADC and 0.2% glycerol) containing 0, 10, 20, 30, 40, and 50 μmol/L of MSM or SMM. Plates were incubated at 37°C, 5% CO_2_, until the growth of colonies. Spontaneous resistant mutants were confirmed by testing their Minimum Inhibitory Concentration (MIC) in liquid media as described above. The genomic DNA from validated mutants was extracted and sent to MicrobesNG for WGS.

### 2.5 Generation of mycobacterial over‐expression constructs

The mycobacterial over‐expression constructs pVV16k‐*ilvB1‐ilvN*, pVV16k‐*ilvN* and pMV261a‐*ilvB1* were generated using standard cloning techniques. Briefly, the WT and mutant genes were amplified from WT and spontaneous‐resistant mutant genomic DNA by PCR (Q5 High Fidelity DNA Polymerase, NEB) using primers described in Table 1 (Eurofins Genomics). The PCR products were cloned into the appropriate vector by exploiting the restriction sites introduced in the primer (as stated in Table 1). All constructs were confirmed by sequencing (Source Biosciences).

**Table 1:** Primers for cloning *ilvB1 *and *ilvN* into mycobacterial expression vectors

Gene	Plasmid	Direction	Primer	Restriction enzyme
*ilvB1*	pMV261a	Forward	CATGCATG**AAGCTT**ATAGCGCACCAACCAAG	*Hin*dIII
		Reverse	CATGCATG**AAGCTT**TCAGGCGTGGCCTTC	
*ilvN*	pVV16k	Forward	GATCCGGAGGAATCACTTC**CATATG**ATGAGCCCGAAGACGCACACGTTGT	*Nde*I
		Reverse	AGTGGTGGTGGTGGTGGTG**AAGCTT**CTTGGCGGTGCCGATGCCGCGCGGA	*Hin*dIII
*ilvB1 + ilvN*	pVV16k	Forward	GATCCGGAGGAATCACTTC**CATATG**GTGAGCGCACCAACCAAGCCACACT	*Nde*I
		Reverse	AGTGGTGGTGGTGGTGGTG**AAGCTT**CTTGGCGGTGCCGATGCCGCGCGGA	*Hind*III

Restriction sites are highlighted in bold type

## 3 RESULTS

### 3.1 Development of high‐throughput screening assay to identify inhibitors of amino acid biosynthesis

**Figure 1:**
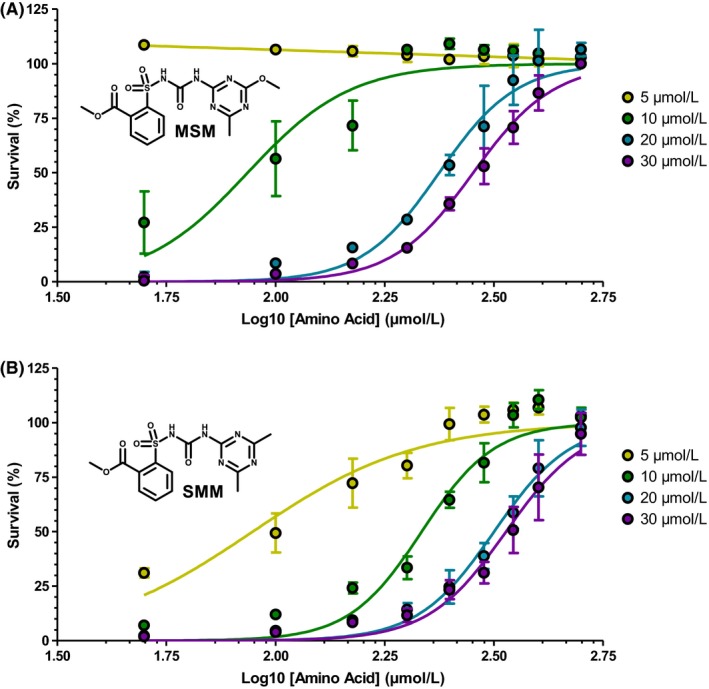
Amino acid supplementation rescues inhibition of *Mycobacterium bovis* BCG by metsulfuron‐methyl (MSM) and sulfometuron‐methyl (SMM). Dose response graph showing *M bovis* BCG rescue from increasing (A) MSM and (B) SMM concentrations with increasing concentrations of all 20 amino acids supplemented into the growth media. Data are displayed as the mean and SD of n = 3. Structures of MSM and SMM are also displayed. The data are colored according to the specified drug concentration

In the TB drug discovery pipeline, identification of the inhibitor target is a fundamental step toward validating the drug potential of a particular compound or scaffold. Target identification commonly involves the WGS of spontaneous‐resistant mutants against compounds that have shown anti‐tubercular activity from a whole throughput screening campaign. There are a number of reported successes when using this approach.^3^, ^19^, ^20^, ^21^ However, to avoid the discovery and progression of compounds that inhibit inappropriate targets, we have developed a new strategy to identify inhibitors of a specific set of biochemical pathways: amino acid biosynthesis. We have designed a high‐throughput phenotypic screen, which can be used to find inhibitors of any amino acid biosynthetic pathway, or tailored to that of a defined amino acid. In this method, we supplemented the growth media with all amino acids, allowing bacteria to survive in the presence of an amino acid biosynthetic inhibitor. To validate this screen, we used MSM and SMM, known inhibitors of branched‐chain amino acid synthesis, which specifically target IlvB1.^22^ *M bovis *BCG was subjected to 5, 10, 20, and 30 μmol/L of MSM and SMM, and supplemented with increasing concentrations of all 20 amino acids up to 500 μmol/L (Figure 1). As the concentration of the amino acids increases, the level of bacterial survival increases indicating that *M bovis *BCG can be rescued from MSM and SMM inhibition with amino acid supplementation.

### 3.2 Identification of the amino acid pathway targeted by an inhibitor

**Figure 2:**
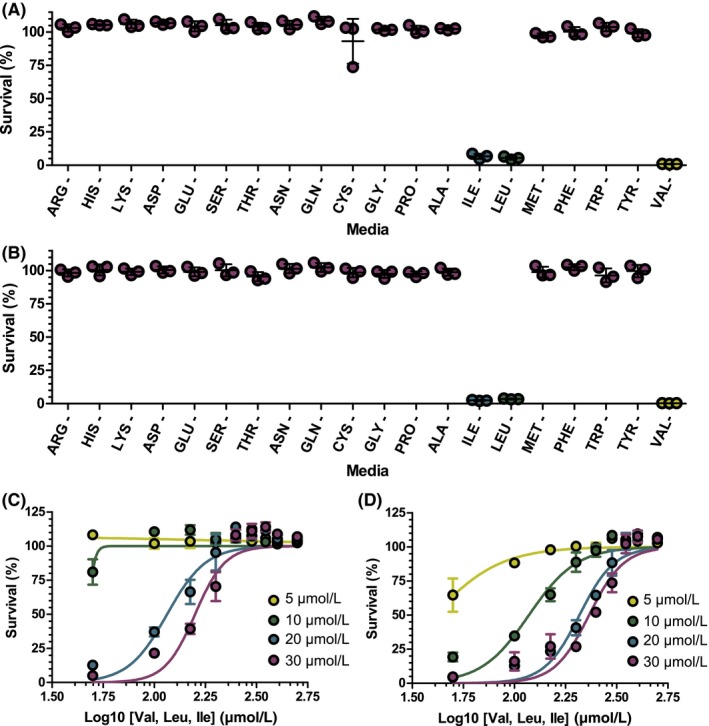
Confirmation of the amino acid biosynthetic pathway targeted by metsulfuron‐methyl (MSM) and sulfometuron‐methyl (SMM) inhibition. A, 50 μmol/L MSM and B, 50 μmol/L SMM inhibition of *M bovis *BCG when supplemented with media containing 1 mmol/L of 19 amino acids. The omitted amino acid is displayed. Each spot is based on a single experiment. (C,D) Dose response graph showing *M bovis* BCG rescue from increasing (C) MSM and (D) SMM concentrations with increasing concentrations of valine, leucine, and isoleucine supplemented into the growth media. The data are colored according to the specified drug concentration. Data are displayed as the mean and standard deviation of n = 3

Following the identification of an inhibitor of amino acid biosynthesis, the specific targeted pathway can be elucidated. By supplementing the growth media with 1 mmol/L of 19 of the 20 amino acids, cell survival can be monitored in the presence of the inhibitor. This was validated by assessing *M bovis* BCG survival using 50 μmol/L of MSM or SMM (Figure 2A, B). When the three branched‐chain amino acids valine, leucine, and isoleucine were individually excluded from the media, the mycobacteria were unable to survive in the presence of the inhibitor. In the individual absence of all other amino acids, the cells were able to survive. The exclusion of proline from the growth media is shown as a control example in Figure 2A, B. It is noteworthy that in this screen, when one branched‐chain amino acid was absent from the media, survival was not possible, indicating that all three amino acids are required to rescue MSM and SMM inhibition. To confirm that only valine, leucine, and isoleucine were required for survival, in the presence of MSM or SMM, the amino acid supplementation was repeated with the three amino acids and full survival was achieved (Figure 2C,D). These results are consistent with the experiments performed by Awasthy and co‐workers.^6^

### 3.3 MSM and SMM target identification

Subsequent to the discovery of an inhibitor of an amino acid biosynthetic pathway, the precise mode of action can then be determined. This can be achieved through a number of techniques, including WGS of resistant isolates, and over‐expression studies of the genes involved in the particular biosynthetic pathway.^3^, ^19^, ^20^, ^21^ Although it is known that the control compounds MSM and SMM target the essential IlvB1 enzyme,^22^ WGS of resistant isolates was performed to identify resistance‐conferring mutations within the gene. This can provide valuable information regarding the inhibitor binding site and for compound optimization.

Initially, the MIC of MSM and SMM was established on solid media: 7.5 and 20 μmol/L, respectively. Spontaneous resistant isolates were raised at 50 μmol/L, and the resistance phenotype was confirmed by MIC determination in liquid media. The gDNA from three mutants per compound was analyzed by WGS and the results are shown in Table 2. As expected, each mutant was found to contain a single‐nucleotide polymorphism (SNP) in *ilvB1 *of greater than 50% mutational frequency within the population. Interestingly, SNPs were also identified in *ilvN*, the regulator of IlvB1 activity. In the six mutant sequences, four different SNPs were identified in *ilvB1*, whereas the same SNP was present in *ilvN*.

**Table 2:** Whole genome sequencing data for *Mycobacterium bovis* BCG spontaneous resistant mutants raised against MSM and SMM. The genomic location of the SNP and the respective gene in which it occurs is stated, along with the codon (the capital letter denotes the base change) and corresponding amino acid residue substitutions. The percentage frequency of the mutation within the gDNA sequenced is stated and corresponds to the frequency of the mutation in the cell population

Compound	Mutant	x MIC MSM	x MIC SMM	Genome position of SNP	Gene	Base change	Amino acid substitution	Mutational frequency (%)
MSM	1	x6.6	x2.5	3361440	*ilvB1*	tGg/tTg	Trp516Leu	100
				3360982	*ilvN*	aCc/aTc	Thr50Ile	100
	2	x6.6	x2.5	3362036	*ilvB1*	gaC/gaA	Asp317Glu	100
				3360982	*ilvN*	aCc/aTc	Thr50Ile	100
	3	x6.6	x2.5	3362791	*ilvB1*	Ccg/Tcg	Pro66Ser	100
				3360982	*ilvN*	aCc/aTc	Thr50Ile	100
SMM	1	x6.6	x2.5	3361440	*ilvB1*	tGg/tTg	Trp516Leu	100
				3360982	*ilvN*	aCc/aTc	Thr50Ile	100
	2	x6.6	x2.5	3362791	*ilvB1*	Ccg/Tcg	Pro66Ser	100
				3360982	*ilvN*	aCc/aTc	Thr50Ile	100
	3	x6.6	x2.5	3362577	*ilvB1*	gTc/gCc	Val137Ala	67.5
				3360982	*ilvN*	aCc/aTc	Thr50Ile	100

MSM, metsulfuron‐methyl; SMM, sulfometuron‐methyl.

**Figure 3:**
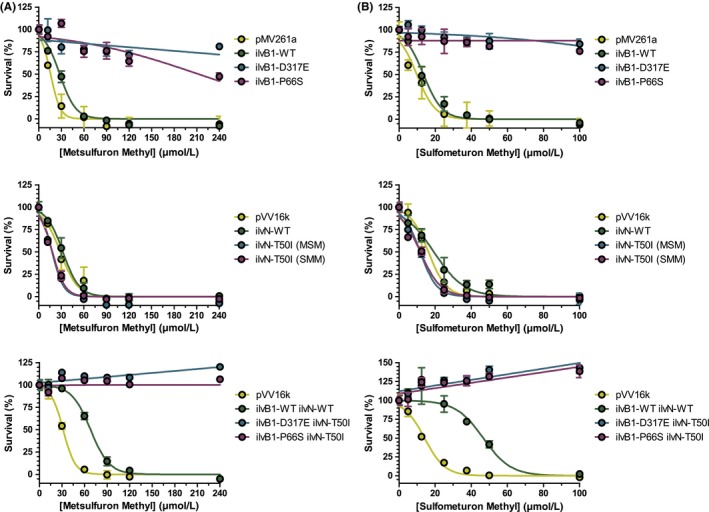
Impact on the MIC of metsulfuron‐methyl (MSM) and sulfometuron‐methyl (SMM) with the over‐expression of IlvB1 and IlvN. The pMV261a and pVV16k constructs containing WT *ilvB1*, *ilvB1*‐D317E, *ilvB1*‐P66S, WT *ilvN*, *ilvN*‐T50I, and combinations of both WT and mutant *ilvB1* with *ilvN* were over‐expressed in *M smegmatis* and the MIC of (A) MSM and (B) SMM was analyzed. Data are displayed as the mean and standard deviation of n = 3

To corroborate that the mutations in *ilvB1 *and *ilvN* were responsible for resistance to MSM and SMM, the impact of over‐expressing *ilvB1 *and *ilvN* on the MIC of the compounds was investigated and the results are shown in Figure 3. WT and mutant versions of *ilvB1* and *ilvN *were cloned into the mycobacterial expression vectors pMV261a and pVV16k, respectively, and the constructs were electroporated into *M smegmatis*. Over‐expression of WT *ilvB1 *and *ilvN* individually showed no improvement in survival when MSM and SMM were added to the medium. Over‐expression of both *ilvB1* and *ilvN* together in pVV16k showed a marginal increase in resistance. However, over‐expression of mutant *ilvB1* resulted in a significant increase in resistance, but over‐expression of mutant *ilvN *alone showed no improvement in the ability to survive in the presence of the compounds. Over‐expression of both mutant genes showed a similar increase in resistance compared to when over‐expressing mutant *ilvB1* alone. These results further show that IlvB1 is the target of MSM and SMM, and the mutations in *ilvN*, although are unable to confer resistance, are likely to be compensatory.

### 3.4 Discovery of inhibitors against amino acid biosynthesis

**Figure 4:**
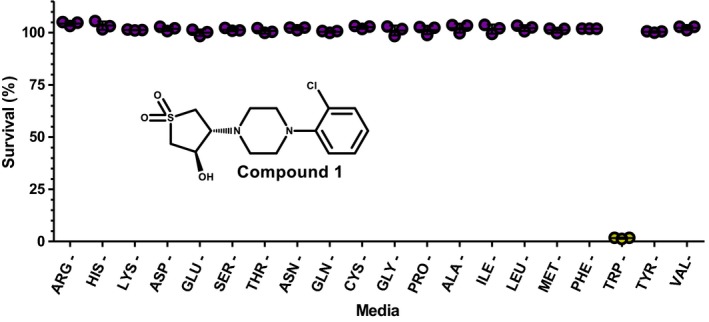
Identification of the amino acid biosynthetic pathway targeted by compound **1**. *Mycobacterium bovis* BCG was challenged with 5 μmol/L compound **1** in media supplemented with 19 amino acids. The mycobacteria were not viable in the absence of tryptophan, indicating the target pathway. The omittance of tryptophan and other amino acids from the supplemented media is shown, where each spot represents a single experiment. The structure of compound **1** is also displayed

In order to benefit from this novel method of identifying inhibitors of amino acid biosynthesis, a GlaxoSmithKline (GSK) compound library consisting of 227 non‐cytotoxic anti‐tubercular compounds (the ‘TB box set’) was screened.^23^, ^24^ With a fixed compound concentration of 20 μmol/L, survival of *M bovis* BCG was established in the presence and absence of 1 mmol/L amino acid supplementation. The amino acid supplementation was shown to rescue cell viability against a single compound, compound **1** (Figure 4). The amino acid pathway targeted by compound **1 **was then elucidated using the method of individual amino acid exclusion from the media. It was discovered that when tryptophan was omitted from the media, *M bovis* BCG was unable to survive in the presence of compound **1**, giving a strong indication that the compound targeted tryptophan biosynthesis (Figure 4). Unexpectedly, the precise mode of action of this compound has previously been elucidated^3^, but the rational and validity of this approach remain evident.

## 4 DISCUSSION

In TB drug discovery, whole‐cell phenotypic high‐throughput screening campaigns of extensive compound libraries have dominated the discovery of anti‐tubercular compounds, which have the potential for progression into drug candidates.^23^, ^24^, ^25^ This current change in strategy from the traditional in vitro enzyme‐specific screens has yielded new inhibitors with unprecedented successes, but not without limitations. With unknown targets, significant amounts of money can be spent pursuing potent inhibitors of unsuitable targets that is, with homologous counterparts in humans. Therefore, understanding the mode of action of an inhibitor is crucial in the early stages of drug discovery, facilitating the progression of medicinal chemistry efforts that turn hits into leads and new drug candidates.

Until recently, target assignment of anti‐tubercular drugs from screening campaigns has typically relied upon the generation and genome sequencing of resistant mutants. Although this remains a validated approach, discovery of new potential inhibitory compound scaffolds has taken a different direction toward phenotypic target‐based high‐throughput screening methods. In this work, we have developed a high‐throughput phenotypic screen to identify inhibitors of amino acid biosynthesis. Amino acid biosynthesis is an example of a promising pathway to target with anti‐tubercular agents, which has currently been underexploited. Amino acids are fundamental to all forms of life. Mycobacteria have the machinery to synthesize all amino acids de novo. The enzymes involved are essential, enabling the cells to survive in the absence of an external source. While mycobacteria can scavenge amino acids from their surroundings in vitro, evidence from auxotroph and transporter studies suggests that at least seven amino acids cannot be scavenged enough in mice or macrophages to compensate auxotrophy.^9^, ^10^, ^11^, ^12^, ^14^, ^16^ In humans, nine amino acids are classified as essential and must be acquired through diet: histidine, isoleucine, leucine, methionine, phenylalanine, threonine, tryptophan, and valine. Targeting the discovery of drugs against these specific biosynthetic pathways would alleviate the concern of a common target in humans, reducing the potential toxicity. Additionally, long‐term oral treatment with inhibitors against amino acid biosynthetic pathways would unlikely effects the gastrointestinal flora, which is rich in amino acids, further protecting against compound toxicity.^7^ These features make amino acid biosynthesis in *Mtb* a plausible route to target in drug discovery, and the essential nature of these processes infers that they also have the potential of broad‐spectrum activities.

**Figure 5:**
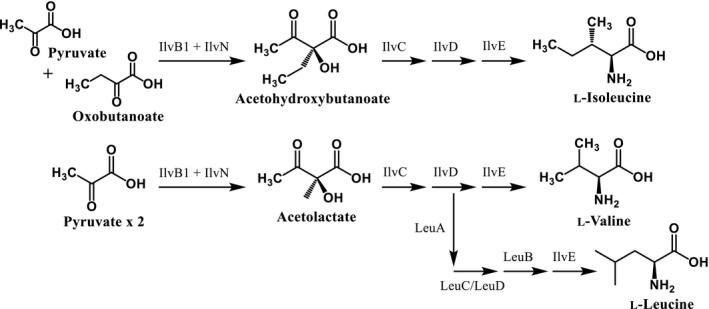
Biosynthetic pathways for branched‐chain amino acids

To discover inhibitors specifically against amino acid biosynthesis, we have optimized a phenotypic screen using known inhibitors of branched‐chain amino acid biosynthesis. Media supplemented with all amino acids have advantages over single amino acid supplementation; for some amino acids, there are multiple routes to their synthesis, and some pathways, such as branched‐chain amino acid biosynthesis, are linked. Therefore, single amino acid supplementation into the media may not provide resistance. For example, as Figure 2A and B show, even with two branched‐chain amino acids in the media, this is not sufficient to rescue survival in the presence of MSM or SMM. Previous studies have confirmed that these inhibitors target the heterotetramer acetohydroxyacid synthase, formed of the catalytic subunit, IlvB1, and the regulatory subunit, IlvN. IlvB1 functions to generate acetolactate from two molecules of pyruvate, precursor to valine and leucine, and also is involved in a separate pathway to generate acetohydroxybutanoate from combining pyruvate and oxobutanoate, leading to the synthesis of isoleucine shown in Figure 5. This is described fully in an excellent review of bacterial branched‐chain amino acid synthesis.^26^ Interestingly, bioinformatic analysis of the *Mtb* genome has revealed that other catalytic subunits exist: *ilvB2*, *ilvG* and *ilvX*; *ilvB2* has low sequence homology with the other three genes. Although it not known whether IlvB2, IlvG, or IlvX are also inhibited by MSM and SMM, WGS analysis only revealed mutations in IlvB1 and IlvN, suggesting that IlvB1 plays the major catalytic role of the acetohydroxyacid synthase activity. Studies investigating the roles of the different catalytic subunits have shown that they are differentially expressed under different physiological conditions, including in stationary phase, hypoxia, and stress conditions.^27^ The survival studies performed in this work were conducted when the cells were in exponential growth phase, where it has been shown that *ilvB1* transcription is elevated compared to in stationary phase.^27^ It is therefore plausible that the other catalytic subunits cannot compensate for IlvB1 inhibition by MSM and SMM due to their differential expression levels during mid‐exponential growth.

It has previously been established that in vitro IlvB1 is the target of MSM and SMM, and that the acetohydroxyacid synthase activity and inhibition is not reliant on the presence of the regulatory subunit, IlvN.^22^ However, the over‐expression studies in this work have demonstrated that increasing the cellular levels of IlvB1 alone is not sufficient to increase the resistance against SMM and MSM, whereas over‐expression of both subunits concomitantly does enable cell survival in the presence of high concentrations of inhibitor. In vivo*,* acetohydroxyacid synthase exists as a heterotetramer, and it is likely that IlvN controls the conformation of IlvB1, regulating its activity. Choi and co‐workers have demonstrated that IlvN does enhance the activity of IlvB1 in vitro. Therefore, over‐expression of IlvB1 alone, although increasing the copy number of the target within the cell, with only native expression levels of IlvN, the conformation may not be suitable to compensate for those complexes inhibited by SMM or MSM, or be in a suitable conformation for inhibition. The activity of the IlvB1 in vitro indicates that the enzyme is in a suitable conformation for activity in the absence of IlvN, which may be a result of the expression of the recombinant protein. Over‐expression of mutant IlvB1 alone did increase the MIC of SMM and MSM. It is likely that the mutated catalytic subunit replaces the WT copies in the heterotetramer, enabling the enzyme to function in the presence of the inhibitors. WT or mutant IlvN over‐expression did not increase the MIC of the inhibitors, suggesting that the inhibitors do not target the regulation of IlvB1 activity and that the mutation in IlvN revealed by WGS is likely to compensate for any conformational changes imparted by the mutations in IlvB1.

Amino acid biosynthesis, although previously suggested as a suitable target for the discovery of anti‐tubercular drugs,^3^, ^7^, ^17^, ^28^ has been largely unexplored. Target‐based screens, be it in vitro kinetic assays or whole cell assays, have been historically exploited to successfully identify inhibitors of a specific enzyme. This method is not without limitations; screening for inhibitors of a particular enzyme eliminates the possibility of discovering inhibitors against other enzymes within the same biochemical pathway. In this work, we have developed a whole‐cell high‐throughput phenotypic screen to discover inhibitors of amino acid biosynthesis. This screen not only ensures compound entry into the cell, but also enables the broad range discovery of inhibitors that can target any point in amino acid biosynthesis and not directed toward a single enzyme. Using the GSK “TB box” set of anti‐tubercular compounds, we identified an inhibitor of tryptophan biosynthesis, which has previous confirmed activity against tryptophan synthase.^3^ This validates the undeniable potential of the screen in the future discovery of inhibitors of amino acid biosynthesis.

## CONFLICT OF INTEREST

The authors declare no competing financial interests.

## AUTHOR CONTRIBUTIONS

Conceived and designed the experiments: C.B., K.A.A., G.S.B. Provided reagents: C.A., J.L. Performed the experiments: C.B., K.A.A., E.J.R. Analyzed the data: C.B., K.A.A., E.J.R., N.J.L., G.S.B. Wrote the paper: C.B., K.A.A., G.S.B.
